# Effect of Intubation Timing on the Outcome of Patients With Severe Respiratory Distress Secondary to COVID-19 Pneumonia

**DOI:** 10.7759/cureus.19620

**Published:** 2021-11-16

**Authors:** Mohamed Fayed, Nimesh Patel, Nicholas Yeldo, Katherine Nowak, Donald H Penning, Felipe Vasconcelos Torres, Abdul Kader Natour, Anoop Chhina

**Affiliations:** 1 Anesthesiology, Pain Management and Perioperative Medicine, Henry Ford Health System, Detroit, USA; 2 Research, Henry Ford Health System, Detroit, USA; 3 Surgery, Henry Ford Health System, Detroit, USA

**Keywords:** kaplan-meier survival curves, sofa score, acute respiratory distress syndrome [ards], high flow nasal canula, rox index, resuscitation, severe respiratory failure, covid-19 respiratory failure, airway intubation, mechanical ventilation

## Abstract

Background

The optimal timing of intubation for critically ill patients with severe respiratory illness remains controversial among healthcare providers. The coronavirus disease 2019 (COVID-19) pandemic has raised even more questions about when to implement this life-saving therapy. While one group of providers prefers early intubation for patients with respiratory distress because these patients may deteriorate rapidly without it, other providers believe that intubation should be delayed or avoided because of its associated risks including worse outcomes.

Research question

Our objective was to assess whether the timing of intubation in patients with severe COVID-19 pneumonia was associated with differences in mortality or other outcomes.

Study design and methods

This was a single-center retrospective observational cohort study. We analyzed outcomes of patients who were intubated secondary to COVID-19 pneumonia between March 13, 2020, and December 12, 2020, at Henry Ford Hospital in Detroit, Michigan. Patients were categorized into two groups: early intubated (intubated within 24 hours of the onset of severe respiratory distress) and late intubated (intubated after 24 hours of the onset of severe respiratory distress). Demographics, comorbidities, respiratory rate oxygenation (ROX) index, sequential organ failure assessment (SOFA) score, and treatment received were compared between groups. The primary outcome was mortality. Secondary outcomes were ventilation time, intensive care unit stay, hospital length of stay, and discharge disposition. Post hoc and Kaplan-Meier survival analyses were performed.

Results

A total of 110 patients were included: 55 early intubated and 55 late intubated. We did not observe a significant difference in overall mortality between the early intubated (43%) and the late intubated groups (53%) (*p* = 0.34). There was no statistically significant difference in patients' baseline characteristics including SOFA scores (the early intubation group had a mean score of 7.5 compared to 6.7 in the late intubation group). Based on the ROX index, the early intubation group had significantly more patients with a reduced risk of intubation (45%) than the late group (27%) (*p* = 0.029). The early intubation group was treated with a high-flow nasal cannula at a significantly lower rate (47%) than the late intubation group (83%) (*p* < 0.001). Significant differences in patient baseline characteristics, treatment received, and other outcomes were not observed. Post hoc analysis adjusting for SOFA score between 0 and 9 revealed significantly higher mortality in the late intubation group (49%) than in the early intubation group (26%) (*p* = 0.03). Patients in the 0 to 9 SOFA group who were intubated later had 2.7 times the odds of dying during hospital admission compared to patients who were intubated early (CI, 1.09-6.67).

Interpretation

The timing of intubation for patients with severe COVID-19 pneumonia was not significantly associated with overall mortality or other patient outcomes. However, within the subgroup of patients with SOFA scores of 9 or lower at the time of intubation, patients intubated after 24 hours of the onset of respiratory distress had a higher risk of death than those who were intubated within 24 hours of respiratory distress. Thus, patients with COVID-19 pneumonia who are not at a high level of organ dysfunction may benefit from early mechanical ventilation.

## Introduction

At the end of 2019, a novel coronavirus was identified as the cause of a cluster of pneumonia cases in Wuhan, China. It rapidly spread, resulting in a global pandemic. This disease has been designated coronavirus disease 2019 (COVID-19) [1], and the virus that causes COVID-19 is severe acute respiratory syndrome coronavirus 2 (SARS-CoV-2). Around 5% of COVID-19 patients develop a critical form of the disease and require intensive care unit (ICU) admission [2,3]. The main manifestation of severe COVID-19 is acute hypoxemic respiratory failure requiring respiratory support, and the optimal supportive treatment for this condition has not yet been established.

The need for mechanical ventilation among patients admitted to the ICU with COVID-19 and respiratory failure varies widely (30-90%), and those patients who receive mechanical ventilation tend to have higher mortality, ranging from 16% to 78% [3-6]. The initial recommendation of the Chinese Society of Anesthesiology Task Force on Airway Management was to proceed with endotracheal intubation for patients showing no improvement in respiratory distress after two hours of high-flow oxygen therapy or other noninvasive support [7,8]. This recommendation was based on the observation that many patients deteriorated precipitously and that they could be more safely intubated at an earlier stage, particularly given the levels of hypoxemia encountered during intubation [7,8]. Few studies at the time had assessed the effect of the timing of intubation and whether it would affect patient outcomes. One systematic review has looked at intubation timing, and it defined early intubation as receiving mechanical ventilation within 24 hours of ICU admission. The synthesized evidence suggested that the timing of intubation based on ICU admission may have no effect on mortality or morbidity of critically ill patients with COVID-19 [9]. However, another study, which included 54 patients, looked at the timing of intubation based on hospital admission, where early intubation was defined as occurring between 4 and 24 hours from admission, while late intubation was defined as intubation from day five to day 10 of admission [10]. They found a mortality benefit in the early intubated group (10). Notably, none of the previous studies used the onset of respiratory distress as time zero for patient enrollment.

The purpose of our study was to analyze the outcomes of patients with severe respiratory distress secondary to COVID-19 pneumonia to see whether patients who received early intubation had different outcomes from those who received late intubation. The primary outcome was mortality, and secondary outcomes were the number of ventilator days, time spent in the ICU, hospital length of stay (LOS), and discharge disposition. Because there are risks associated with delaying intubation, we hypothesized that patients who received early mechanical ventilation would have lower mortality, morbidity, and hospital LOS than those who were intubated later in their hospitalization.

## Materials and methods

We conducted a single-center retrospective chart review study. The population consisted of patients with COVID-19 pneumonia confirmed with a positive polymerase chain reaction test that developed into severe respiratory distress, who were admitted to the Henry Ford Hospital in Detroit, Michigan, a level 1 trauma tertiary care center, between March 13, 2020, and December 12, 2020. We received approval from the institutional review board at the Henry Ford Health System stating that this research was appropriate in design and that it met the requirements of the Federal Guidelines, 45 CFR Part 46 and 21 CFR Part 50 (approval number 14370). Eligible patients were identified using the Henry Ford Health System EPIC electronic medical record database. Patients included in the analysis had COVID-19 pneumonia with bilateral infiltrates indicated by chest X-ray in addition to at least one of the following criteria: (1) respiratory rate >30 for at least two hours and (2) oxygen saturation <93% for at least two hours. Patients who had a “do not intubate” order at the time of enrollment were excluded from the analysis. The 110 eligible patient charts were divided into two groups: an early intubated group (intubated within 24 hours of meeting inclusion criteria) and a late intubated group (intubated after 24 hours of meeting inclusion criteria).

We looked at patient baseline characteristics, including age, body mass index (BMI, expressed as the ratio kg/m2), history of diabetes mellitus, chronic obstructive pulmonary disease, chronic kidney disease, ischemic heart disease, and sequential organ failure assessment (SOFA) score (at time of intubation). We assessed the respiratory rate oxygenation (ROX) index as a measure of severity of respiratory distress at the time of enrollment. We subsequently classified patients into low and high risk of intubation based on the ROX index. We also evaluated the incidence of acute renal failure during hospitalization (defined per the RIFLE [risk, injury, failure, loss of kidney function, and end-stage kidney disease] criteria, with either an increase in creatinine that is three times the baseline or a creatinine level >4 mg/dL) and whether there was a need for hemodialysis. We investigated whether patients were treated with steroids (prednisone, methylprednisone, or dexamethasone), remdesivir, a high-flow nasal cannula (HFNC) prior to intubation, muscle relaxants (cis-atracurium or rocuronium infusion) during mechanical ventilation, and the use of tidal volume (TV) while on mechanical ventilation.

The primary outcome was patient mortality. Secondary outcomes were ventilation days, days spent in the ICU, hospital LOS, and discharge disposition. Discharge disposition categories were as follows: discharged home to self-care (routine discharge), discharged home to care of organized home health service, discharge to certified long-term care hospital, discharge to a skilled nursing facility, and other discharge.

We also looked into respiratory failure as the cause of death in both groups. Based on current evidence [11], there are two classifications for pulmonary contribution to death. First, a severe pulmonary disease, defined as “inability to liberate from mechanical ventilation, non-invasive ventilation, or heated high flow nasal cannula due to inadequate oxygenation or ventilation without aforementioned support.” Another one is an irreversible pulmonary disease, which is defined as “insupportable oxygenation or ventilation (defined as PaO2 <40 mmHg on FIO2 1.0 for >2 h or respiratory acidosis with pH <7.1 on maximum ventilator settings) as well as if care was withdrawn due to poor prognosis related to pulmonary organ system dysfunction.”

Numeric variables were summarized with mean and standard deviation (SD) or median and interquartile range (IQR) and were compared using independent t-test or Mann-Whitney U tests. Categorical variables were summarized with frequencies and proportions and were compared using the chi-square test or Fisher's exact test. A post hoc comparison was conducted for patients with SOFA scores between 0 and 9. All tests were two-tailed. Kaplan-Meier survival analysis was done to compare the long-term survival of patients having a SOFA score equal to or less than 9 to those having a SOFA score greater than 9. Pairwise comparisons within each SOFA score stratum were performed using the log-rank (Mantel-Cox) test. A p-value <0.05 was considered statistically significant.

## Results

Patient characteristics

A total of 110 patient charts were included in this analysis, including 55 patients who received early intubation and 55 patients who received late intubation (Table 1). Apart from the ROX index, no statistically significant differences in baseline characteristics were observed (Table 1). The mean age was 63.9 years (SD, 14.1) and the median BMI was 30.4 (IQR, 26.0-38.1), with no significant difference in either variable (age, p = 0.762; BMI, p = 0.317). The early intubation group had a higher, but not significantly different, median SOFA score (7.5; IQR 5-10) than the late intubation group (6.71; IQR, 5-8) (p = 0.057). The early intubation group had a higher mean ROX index (5.4; SD, 3.4) than the late intubation group (4.4; SD, 2.5). Based on the ROX index, 45% of patients in the early intubation group and 27% of patients in the late intubation group were classified as having a reduced risk of intubation, and overall ROX index risks differed between groups (p = 0.029) (Table 1).

**Table 1:** Baseline characteristics of patients who required mechanical ventilation secondary to severe COVID-19. BMI, body mass index; CI, confidence interval, CKD, chronic kidney disease; COPD, chronic obstructive pulmonary disease; IHD, ischemic heart disease; IQR, interquartile range; ROX, respiratory rate oxygenation index; SOFA, sequential organ failure assessment; SD, standard deviation.

Variable	All (N = 110)	Early intubated (n = 55)	Late intubated (n = 55)	p-value
Age, year, mean (SD)	63.9 (14.1)	64.3 (12.7)	63.5 (15.5)	0.762
BMI, kg/m^2^, median (IQR)	30.4 (26.0-38.1)	29.5 (24.4-38.8)	31.0 (27.1-37.5)	0.317
IHD, n (%)	10 (9.1)	5 (9.1)	5 (9.1)	1.000
Diabetes, n (%)	52 (51)	23 (48.9)	29 (52.7)	0.703
COPD, n (%)	20 (19.6)	11 (23.4)	9 (16.4)	0.372
Acute renal failure, n (%)	75 (68.2)	36 (65.5)	39 (70.9)	0.539
Dialysis, n (%)	35 (31.8)	17 (30.9)	18 (32.7)	0.838
CKD, n (%)	71 (69.6)	33 (70.2)	38 (69.1)	0.902
ROX score, mean (SD)	4.9 (3.0)	5.4 (3.4)	4.4 (2.5)	0.082
Risk of intubation, n (%)				0.029
Reduced risk	40 (36.4)	25 (45.5)	15 (27.3)	
Intermediate risk	11 (10.0)	2 (3.6)	9 (16.4)	
High risk	59 (53.6)	28 (50.9)	31 (56.4)	
SOFA score, mean (IQR) [95% CI]	7.1 (5-9) [6.6-7.6]	7.5 (5-10) [6.7-8.3]	6.7 (5-8) [6.1-7.3]	0.057

Treatments for COVID-19

**Figure 1:**
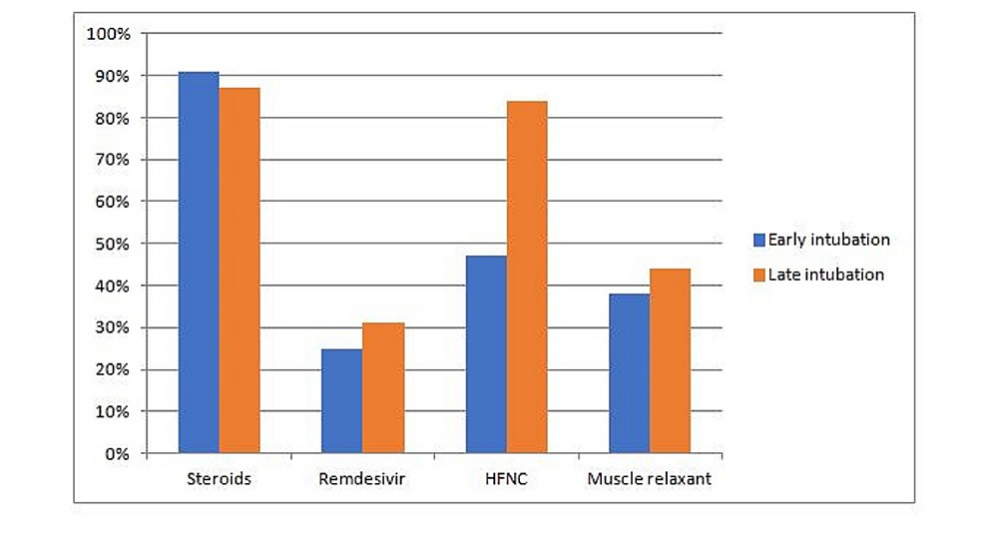
Treatments received by patients who required mechanical ventilation secondary to severe COVID-19. HFNC, high-flow nasal cannula.

Steroids were given to the majority of patients (overall, 89%; early intubated, 90.9%; late intubated, 87.3%; p = 0.54). Steroids were started when patients needed supplemental oxygen, and steroid treatment did not change with intubation timing. Remdesivir was given to 31% of all patients, and there was no difference in remdesivir use between the groups (early intubated, 25%; late intubated, 30%; p = 0.525). HFNC was used for 65% of all patients and was used significantly more in the late intubation group (early intubated, 47% versus late intubated, 84%; p < 0.001) (Figure 1). A total of 45 patients received a muscle relaxant infusion while being on mechanical ventilation, with 21 patients (38%) in the early intubation group receiving a muscle relaxation infusion for a mean of 3.1 days (SD, 1.9) and 24 patients (44%) in the late intubation group receiving a muscle relaxant infusion for a mean of 2.6 days (SD, 2.2) (Figure 1). There was no significant difference in the use of muscle relaxants between the two groups (p = 0.228). No significant difference in the use of TV was observed between groups. The early ventilation group had mean TV of 6.3 mL/kg of predicted body weight (SD, 1.3), and the late ventilation group had a mean TV of 5.9 mL/kg of predicted body weight (SD, 1.0) (p = 0.125).

Main outcomes

Of the 110 patients, 53 died (48%), including 24 (44%) patients who were intubated early and 29 (53%) patients who were intubated late (p = 0.34); thus, overall mortality between the groups was not significantly different. The median ventilation time for all patients was 12.5 days (IQR, 7-22), and median ventilation time did not significantly differ between early intubated (14 days; IQR, 10-23) and late intubated patients (10 days; IQR, 4.5-21; p = 0.084). The median number of days spent in the ICU for the entire cohort was 16 days (IQR, 10-23) (early intubated, 17 days; IQR, 11-26 vs. late intubated, 15 days; IQR, 11-27; p = 0.948). The median hospital LOS for the group was 18.5 days (IQR, 13-29) (early intubated, 18 days; IQR, 11-30 vs. late intubated, 19 days; IQR, 14-29; p = 0.428). Of the four patients who were discharged to home self-care (7%), one patient (3.2%) had been intubated early and three patients (12%) had been intubated late (p = 0.297) (Table 2). As a cause of mortality due to pulmonary disease, our entire patients in both groups had severe pulmonary disease criteria at the time of death. Regarding irreversible pulmonary disease, there was no statistically significant difference in both groups where it contributed to a total of 19 out of 24 deaths (79%) in the early intubation group and 19 out of 29 deaths (65%) in the late intubation group (p = 0.27).

**Table 2:** Primary and secondary outcomes of patients who received mechanical ventilation secondary to severe COVID-19. ICU, intensive care unit; IQR, interquartile range; LOS, length of stay.

Outcome	All (N = 110)	Early intubated (n = 55)	Late intubated (n = 55)	p-value
Mortality, n (%)	53 (48.2)	24 (43.6)	29 (52.7)	0.340
Ventilation time, median (IQR), days	12.5 (6.8-22.0)	14 (10-22.5)	10 (4.5-20.5)	0.084
ICU stay, median (IQR), days	16 (10.0-26.3)	17 (10.5-26)	15 (10.5-27)	0.948
Hospital LOS, median (IQR), days	18.5 (12.8-29.3)	18 (10.5-29.5)	19 (14-28.5)	0.428
Discharge disposition				0.297
Home under self-care, n (%)	4 (7.0)	1 (3.2)	3 (11.5)	
Home under the care of organized home health service org, n (%)	16 (28.1)	8 (25.8)	8 (30.8)	
Medicare-certified long-term care hospital, n (%)	11 (19.3)	5 (16.1)	6 (23.1)	
Skilled nursing facility, n (%)	22 (38.6)	13 (41.9)	9 (34.6)	
Other, n (%)	4 (7.0)	4 (12.9)	0 (0)	

Post hoc analysis of patients with SOFA scores between 0 and 9 and multivariate analysis

Baseline Characteristics

Post hoc analysis showed that 89 patients had SOFA scores between 0 and 9 (38 in early intubation and 51 in late intubation) (Table 3). Of these patients, median SOFA scores were 5.9 for the early intubation group (IQR, 4.3-7.0) and 6.3 for the late intubation group (IQR, 5.0-7.0), with no significant difference between groups (p = 0.14). ROX index within this patient subset did not differ significantly between groups, where 50% of patients in the early intubation group and 55% in the late intubation group were at high risk of intubation (p = 0.059).

**Table 3:** Post hoc analysis of patients with SOFA scores 0-9 (N = 89), baseline characteristics. CI, confidence interval, IQR, interquartile range; ROX, respiratory rate oxygenation; SOFA, sequential organ failure assessment.

Variable	Early intubated (n=38)	Late intubated (n=51)	p-value
Age, mean (SD), years	62.6 (12.8)	63.4 (15.9)	0.40
BMI, mean (SD) kg/m^2^	32.4 (9.7)	33.7 (9.4)	0.26
Acute renal failure, n (%)	22 (57.9)	37 (51.4)	0.15
Dialysis, n (%)	9 (24)	15 (29)	0.5
ROX index			0.059
Low risk for intubation n (%)	18 (47)	15 (29)	
Intermediate risk for intubation n (%)	1 (3)	8 (16)	
High risk for intubation n (%)	19 (50)	28 (55)	
SOFA score median, (IQR) [95% CI]	5.9 (4-7) [5.7-6.6]	6.3 (5-7) [5.8-6.7]	0.14

Outcomes

Mortality was significantly higher in patients who had been intubated late (49%) than in those who had been intubated early (26%) (p = 0.03). While patients in the early intubation group were intubated for a slightly longer time than those in the late intubation group (17 vs. 10 days; p = 0.055), the difference was not significant. There were no significant differences between groups in any other secondary outcome (Table 4). Lastly, multivariate analysis showed that patients in the late intubation group were approximately 2.7 times (odds ratio) more likely to die compared to patients in the early intubation group (95% CI, 1.09-6.67) (Table 5). There was no statistically significant difference in the final cause of death in both groups, where 8 out of 10 patients (75%) in the early intubation group and 17 out of 25 patients (68%) in the late intubation group died from irreversible pulmonary disease (p = 0.48).

**Table 4:** Post hoc analysis of patients with SOFA scores 0-9 (N = 89), outcomes. IQR, interquartile range; LOS, length of stay; SOFA, sequential organ failure assessment.

Variable	Early intubated (n = 38)	Late intubated (n = 51)	p-value
Mortality, no. (%)	10 (26.3)	25 (49.0)	0.03
Ventilation time, median (IQR), days	17 (10-25)	10 (5-22)	0.055
ICU stay, median (IQR), days	19 (13-28)	17 (11-28)	0.438
Hospital LOS, median (IQR), days	23 (13-31)	19 (14-30)	0.826
Discharge disposition			0.272
Home under self-care, n (%)	1 (3.6)	3 (11.5)	
Home under the care of organized home health service org, n (%)	8 (28.6)	8 (30.8)	
Medicare-certified long-term care hospital, n (%)	4 (14.3)	6 (23.1)	
Skilled nursing facility, n (%)	11 (39.3)	9 (34.6)	
Other, n (%)	4 (14.3)	0 (0.0)	

**Table 5:** Multivariate analysis of mortality.

	Odds ratio (95% CI)	p-value
Early intubated	1.0 (Reference)	
Late intubated	2.69 (1.09-6.67)	0.032

Kaplan-Meier survival analysis

**Figure 2:**
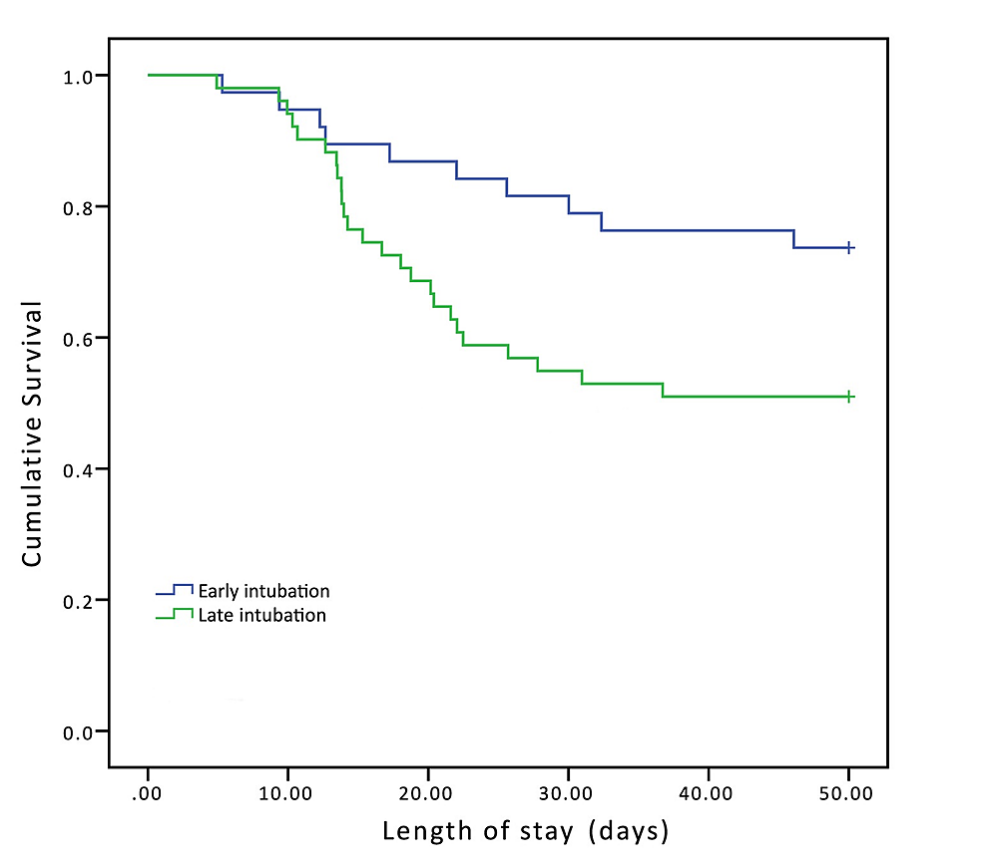
Kaplan-Meier curve for patients with SOFA score 0-9. Blue line = early intubation group and green line = late intubation group. SOFA, sequential organ failure assessment.

**Figure 3:**
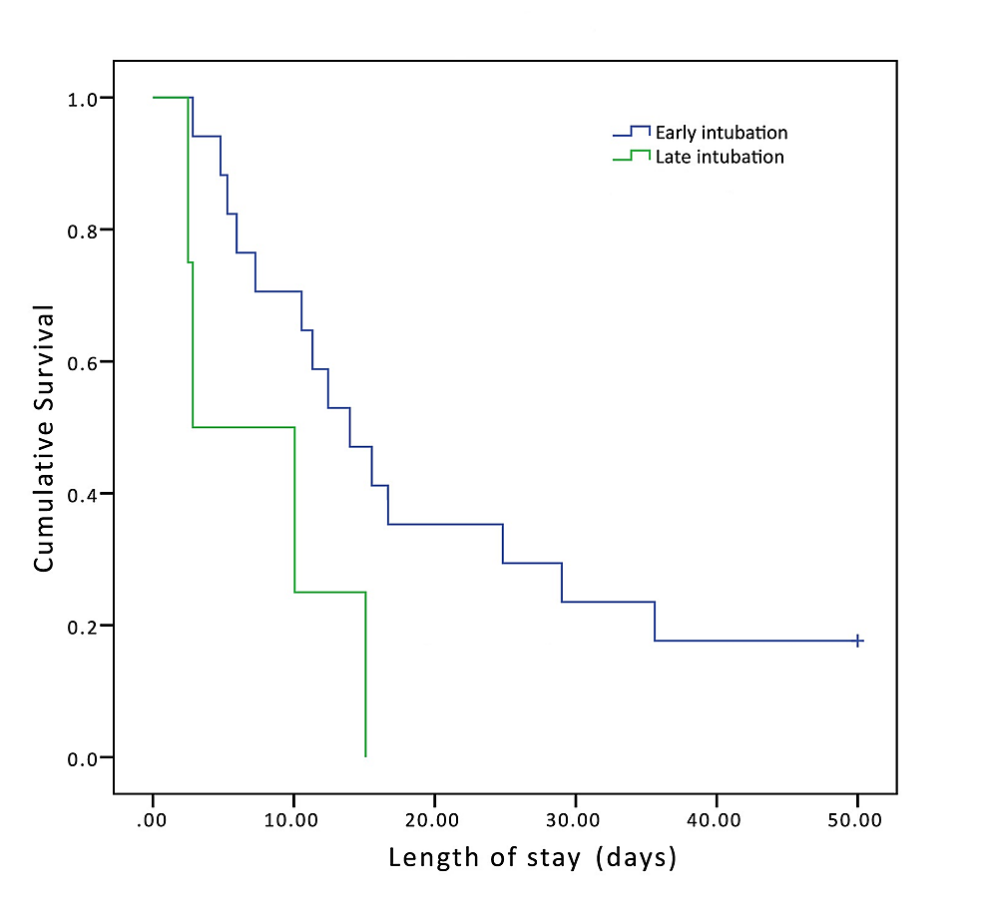
Kaplan-Meier curve for patients with SOFA score >9. Blue line = early intubation group and green line = late intubation group. SOFA, sequential organ failure assessment.

Figures 2, 3 show Kaplan-Meier survival analysis comparing patients with respiratory failure secondary to COVID-19 who underwent early (blue, n = 55) versus late (green n = 55) intubation. Time to death was measured from the time of hospital admission. Patients were stratified into two groups based on SOFA score at the time of intubation: SOFA score 0-9 (Figure 2) and SOFA greater than 9 (Figure 3). Of the patients in the early intubation group, 38 patients had a SOFA score of 0-9 and 17 patients had a SOFA score greater than 9. Of the patients in the late intubation group, 51 patients had a SOFA score of 0-9 and only four patients had a SOFA score greater than 9. Pairwise comparisons within each stratum showed that early intubation was significantly associated with higher survivability (log-rank test, p = 0.028 for SOFA 0-9 and p = 0.039 for SOFA >9).

## Discussion

In this study, we did not observe significant differences in overall mortality, ventilation days, ICU and inpatient LOS, or discharge disposition between patients who were intubated early versus late for severe pneumonia secondary to COVID-19. SOFA scores were slightly but not significantly higher in the patients who were intubated early than in those who were intubated later. However, post hoc analysis of patients who had SOFA scores between 0 and 9 showed that significantly more patients within this score range who were intubated late died compared to those who were intubated early. Additionally, Kaplan-Meier survival analysis stratified by SOFA score also showed that patients who were intubated later were more likely to die. It is worth noting that a higher number of patients in the late intubation group were in SOFA scores between 0 and 9 and compared to 38 patients in the early intubation group. Apart from the use of HFNC, there were no significant differences in the treatments received by each group, and HFNC had been used in the majority of patients in the late intubation group. This may partially explain why intubation was delayed for these patients, as the treatment team may have waited to see if the HFNC would be helpful.

Overall SOFA scores alone may not provide an adequate explanation for the difference we observed in mortality between the intubation groups. The original SOFA study showed that critically ill patients (not including COVID-19) with an initial SOFA score of 9 or lower had a mortality rate of less than 33%, while patients with scores over 11 had a predicted mortality rate of 95% [12], indicating that higher scores are associated with higher mortality. In our analyses, by stratifying patients based on SOFA scores, we saw that the timing of intubation was associated with a mortality difference. For patients who had SOFA scores indicating a potentially higher risk of mortality (>9), outcomes were predictably not favorable for either intubation group, but patients who had been intubated later fared the worst. Interestingly, analysis of the subset of patients in the 0 to 9 SOFA score group, which generally indicates a lower risk of mortality, still showed that those patients who were intubated later were significantly more likely to die. We did not appreciate any difference in baseline characteristics in the subset of patients with SOFA scores 0-9 to explain this mortality difference apart from the ROX index. Even though our study did not show a statistically significant difference in ROX index between groups, the early intubation group trended toward a higher percentage of patients with a low risk of intubation compared to the late intubation group and both groups had almost the same percentage of high risk for intubation. It is possible that the early intubation group may have had a larger percentage of patients with better respiratory reserve than the late intubation group, and hence, could tolerate intubation with less risk of decompensation, including hypoxemia. Note that all of our patients were treated in the same institution with similar guidelines for managing COVID-19, including early proning and lung-protective ventilation and treatment protocols. This was evident in that the use of TV during mechanical ventilation did not differ between groups as well as other treatments as steroids, remdesivir, and muscle relaxant use. Our findings suggest that COVID-19 patients who do not exhibit extreme organ dysfunction as indicated by SOFA score, and even those patients with ROX index indicating low risk of intubation, still may benefit from mechanical ventilation soon after exhibiting severe respiratory distress rather than later.

The question of when to use mechanical ventilation for patients with COVID-19 pneumonia is challenging, and it is particularly complex in the early phase of illness when patients may have normal lung mechanics, which could sway physicians away from choosing ventilation [13-15]. But intubating patients early may have several benefits. First, there is evidence that patients with COVID-19 pneumonia have severe hypoxemia with large TVs (14) that could lead to more patient self-inflicted lung injury [7,16-18]. Thus, experts have argued that protective mechanical ventilation with effective sedation and paralysis should be implemented early to prevent the subsequent risk of volutrauma and patient self-inflicted lung injury due to large respiratory effort during noninvasive ventilatory support [14]. However, intubating COVID-19 patients may take more time than what is required in a standard intubation setting, since time is needed for the intubation team to assemble and don appropriate personal protective equipment and for transferring the patient to an appropriate location while maintaining infection control measures. These delays due to complex procedures could lead to a prolonged period of hypoxemia, and patients might subsequently deteriorate before receiving mechanical ventilation, resulting in increased morbidity and mortality. Another potential advantage of early intubation rather than using HFNC or noninvasive ventilation is that it can reduce the risk of viral particle aerosolization and viral exposure of healthcare workers [5,19].

Notably, mechanical ventilation is not a risk-free treatment modality and is inherently associated with a number of well-described complications such as ventilator-associated pneumonia [20], ventilator-induced lung injury [21], hemodynamic disturbances [22], and issues related to sedation and immobilization [23]. Therefore, the decision to put COVID-19 patients on mechanical ventilation is not trivial, and more research is needed so that we can improve guidelines for when to intubate patients with COVID-19 for optimal outcomes.

Our study has several limitations. It is a retrospective chart review with a low patient population; therefore, results may not be generalizable. Also, the decision to treat patients with mechanical ventilation was based on physician discretion, and treatment guidelines for COVID-19 were in a state of flux during the study period.

## Conclusions

We observed no significant differences in overall mortality or other outcomes between patients with severe COVID-19 who were intubated early after presenting with severe respiratory illness and those who were intubated later. However, in the subset of patients who had lower SOFA scores at the time of intubation, patients who were intubated after 24 hours of the onset of respiratory distress were more than two and a half times more likely to die during hospitalization than patients who were intubated earlier.

Our findings suggest that patients with severe respiratory distress secondary to COVID-19 pneumonia who do not have multi-organ failure may benefit from earlier treatment with mechanical ventilation rather than non-invasive respiratory therapies. Larger, controlled studies, systematic reviews, or meta-analyses are needed to elucidate the best timing strategy for treating patients who have severe COVID-19 with mechanical ventilation.
